# On Tuberculous Disease of the Lymphatic Glands—I

**Published:** 1910-03-19

**Authors:** 


### SURGERY.

### ON TUBERCULOUS DISEASE OF THE LYMPHATIC GLANDS—I.

No set of lymphatic glands in the body is immune from tuberculous infection. Some, however, are commonly attacked, but others very infrequently. Tuberculous disease of the lymphatic glands of the neck is one of the commonest conditions met with in surgery. Another set which is more frequently infected than the textbooks of surgery would lead one to suppose is the inguinal chain of glands. In any unexplained enlargement of these glands the possibility of tuberculosis should be kept in mind. By " unexplained " is meant an enlargement where no primary septic or venereal process can be discovered on the genitalia or in the inguinal region. The diagnosis then lies between gumma on the one hand and tubercle on the other. In the absence of a history of past syphilis or if the condition fails to react to the exhibition of potassium iodide, the enlargement is most probably tuberculous in origin.

A tuberculous retropharyngeal abscess is commonly described as being due to tuberculous infection of a chain of lymphatic glands which lie in front of the cervical vertebræ in children, but tend to disappear as adult life is reached. This statement appears to have been copied blindly from one textbook to another without critical examination. There is, for instance, little or no proof that any chain of glands behaving in such an abnormal manner exists at all in this region. It seems much more reasonable to suppose that such abscesses are due either to a patch of caries on the anterior aspect of one or more of the cervical vertebræ or simply to infection of the cellular tissue which lies between the posterior wall of the pharynx and the spinal column. This, however, by the way.

What is the explanation of the disproportionate frequency of tuberculous infection of the cervical set? It is due to the fact that they drain the mouth and pharynx, a cavity which is particularly liable to a variety of inflammatory conditions. The tonsils, for instance, are often enlarged and chronically inflamed, and dental caries is of very common occurrence. Either of these conditions acts as a chronic irritant and thus throws work on the lymphatic glands of the neck, which impairs their vitality and diminishes their power of resisting invasion. Further than this, it has been shown that the tonsil itself is one of the channels whereby the tubercle bacillus enters the system. The respiratory tract is, of course, the other chief source of infection. Now if the tonsil is healthy it is supposed to act as a filter, which entraps and deals with inspired pathogenic micro-organisms; but if it is diseased it easily falls a prey; and though there may be no macroscopic evidence of tuberculous disease of the tonsil itself, since a tuberculous tonsillitis is comparatively rare, the tubercle bacilli get past the first line of defence and invade the nearest lymphatic glands.

For a complete understanding of the clinical aspects of tuberculous glands in the neck, some knowledge of the anatomy of these structures is essential. Roughly speaking, there are three important sets of glands, other than the deep chain which surrounds the main vessels. These are (i.) a submaxillary group, lying, as their name implies, in close connection with the submaxillary salivary gland in the digastric triangle. They drain the anterior part of the face, the mucous membrane of the mouth, and the anterior part of the tongue. This is generally the first set attacked in epithelioma of the tongue; (ii.) a sublingual set lying under cover of the ramus of the mandible on the mylo-hyoids, and internal on each side to the anterior belly of the digastric muscle. They drain the anterior part of the mucous membrane of the floor of the mouth and are the first to enlarge, if an epithelioma starts in that situation; and (iii.) a more superficial set, some of which lie in front of the external auditory meatus and others along the anterior border of the upper part of the sternomastoid muscle.

The remaining glands are often classed together as one group, known as the concatenate chain, occupying a vertical position in the neck in close relation to the big vessels, but these again may be subdivided into four subsidiary groups, as has been pointed out by Mr. Harold Stiles in Mr. Burghard's " System of Operative Surgery." According to him, this chain may be divided into two groups situated respectively above and below the omo-hyoid muscle, and each of these is again subdivided into an anterior and posterior. Of these the upper and anterior group is generally attacked first by tuberculosis because it drains the tonsil and pharynx directly. But the infection may travel from any of these separate groups to others because all are interconnected with afferent and efferent lymphatic vessels.

Knowing these anatomical facts, one will be able to recognise how many of the groups are affected and in what way the infection is likely to travel, if it is not checked. In considering operative treatment for tuberculous enlargement, it is important to know which group is mainly involved, since the incision will vary in each case. The mistake that is most often made is to make an incision which cannot be adequately enlarged so as to allow of extirpation of all the affected glands without making an unsightly scar. There are few conditions in surgery in which so good a result can be obtained by thorough operative treatment or one so unsatisfactory if an inadequate operation is performed.

